# Graph-based active learning of agglomeration (GALA): a Python library to segment 2D and 3D neuroimages

**DOI:** 10.3389/fninf.2014.00034

**Published:** 2014-04-04

**Authors:** Juan Nunez-Iglesias, Ryan Kennedy, Stephen M. Plaza, Anirban Chakraborty, William T. Katz

**Affiliations:** ^1^FlyEM Project, HHMIAshburn, VA, USA; ^2^Department of Computer and Information Science, School of Engineering and Applied Sciences, University of PennsylvaniaPhiladelphia, PA, USA; ^3^Video Computing Group, Department of Electrical Engineering, University of California at RiversideRiverside, CA, USA

**Keywords:** connectomics, Python, electron microscopy, image segmentation, machine learning

## Abstract

The aim in high-resolution connectomics is to reconstruct complete neuronal connectivity in a tissue. Currently, the only technology capable of resolving the smallest neuronal processes is electron microscopy (EM). Thus, a common approach to network reconstruction is to perform (error-prone) automatic segmentation of EM images, followed by manual proofreading by experts to fix errors. We have developed an algorithm and software library to not only improve the accuracy of the initial automatic segmentation, but also point out the image coordinates where it is likely to have made errors. Our software, called gala (graph-based active learning of agglomeration), improves the state of the art in agglomerative image segmentation. It is implemented in Python and makes extensive use of the scientific Python stack (numpy, scipy, networkx, scikit-learn, scikit-image, and others). We present here the software architecture of the gala library, and discuss several designs that we consider would be generally useful for other segmentation packages. We also discuss the current limitations of the gala library and how we intend to address them.

## 1. Introduction

Connectomics, the elucidation of complete neuronal circuits, requires resolutions as low as 5–10 nm to distinguish the smallest neuronal processes, but also fields of view hundreds of micrometers across or more, as neurons can easily span those distances. This size disparity results in large image volumes of at least 10 gigavoxels and often orders of magnitude larger. Neurons are visible as distinct regions, or segments, in this 3-dimensional image volume. Combining an accurate segmentation with the position of pre- and post-synaptic sites in the image (Kreshuk et al., 2011; Jagadeesh et al., in press), one can obtain the shapes, locations, and connectivity of all the neurons in an image volume, as has been demonstrated in Helmstaedter et al. (2013) and Takemura et al. (2013).

Various methods have been proposed (and implemented) to go from images to neuronal morphology and connectivity. Cardona et al. (2010) used manual tracing of the midline of neuronal processes, along with manual annotation of synapses, to analyze a neuronal circuit. Helmstaedter et al. (2011) refined this approach by having multiple individuals trace the same neuron, thus allowing them to estimate the error rate of their traces. Manual tracing alone, however, will not scale to the reconstruction of large neuronal circuits (Helmstaedter, 2013). An alternate strategy, then, has been to use automatic segmentation of the neurons in the image volume, followed by human proofreading of this segmentation (Chklovskii et al., 2010). Until recently, this proofreading was the rate-limiting step for neuronal reconstruction from EM images, for two reasons: first, the segmentation algorithms were (and still are) orders of magnitude too inaccurate to reconstruct even a single neuron without errors; and second, the human proofreaders had to examine every voxel of the image, even if the automatic segmentation is correct. In response, we developed a new machine learning-based algorithm for image segmentation (Nunez-Iglesias et al., 2013) that provides state of the art automatic segmentation accuracy and then directs proofreaders to likely areas of error in the segmentation. This has dramatically sped up proofreading and reconstruction speed (5–16-fold, in our anecdotal observations).

The algorithm, called GALA (graph-based active learning of agglomeration), works by repeatedly consulting a gold standard segmentation (prepared by human annotators) as it agglomerates sub-segments according to its current best guess. (Note: throughout this paper, we will use “GALA” to describe the algorithm, and “gala” or “Gala” for the Python library and software.) It thus accumulates a training dataset used to fit a classifier, which guides subsequent agglomeration decisions. Furthermore, through the probability output of the classifier, it can estimate its own confidence in whether two segments should be merged, and this estimate can be used for proofreading.

**Figure 1:**
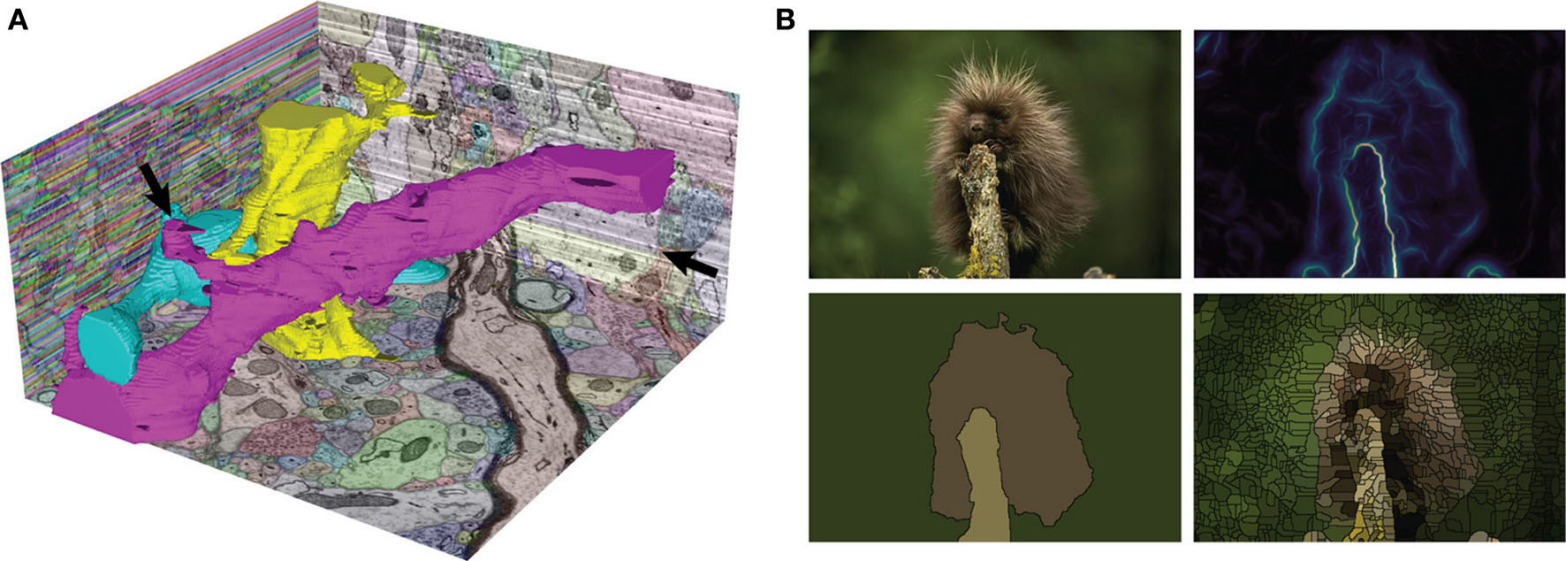
**Two sample automatic segmentations performed with gala. (A)** The SNEMI3D test data. The XZ plane (left) shows the initial, extremely oversegmented superpixel map, while the YZ (right) and XY (bottom) planes show the final segmentation returned by gala. Three complete neuronal segments are highlighted in 3D. Note that the segmentation is not perfect—stubs on the magenta 3D segment are candidates for missed branches, and a big false split is apparent on the YZ cut plane (arrows). 3D shape features should improve these results (Bogovic et al., 2013). **(B)** Our favorite fuzzball from the Berkeley Segmentation Data Set. Clockwise from top-left: original image, gPb boundary probability map (using the cubehelix colormap), watershed superpixels, and final GALA segmentation using threshold of 0.5.

GALA outperformed previous agglomeration methods for automatic segmentation of an isotropic (10 × 10 × 10 nm resolution) focused ion beam scanning electron microscope (FIBSEM) dataset of *Drosophila* larva neuropil (Nunez-Iglesias et al., 2013). What's more, it remained top-ranked for 8 months in the SNEMI3D challenge, which uses anisotropic neuronal EM data of 6 × 6 × 30 nm resolution, against tools developed specifically for anisotropic data and comprising about 70 submissions (Liu et al., 2012; Arganda-Carreras et al., 2013; Kaynig et al., 2013; Liu et al., 2014). Finally, thanks to the nD design of gala, we were able to segment 2D natural images and outperform state of the art agglomerative methods on the Berkeley Segmentation Data Set (BSDS) (Nunez-Iglesias et al., 2013). See Figure 1 for two segmentation examples obtained with the gala library.

Since we have described the details of the GALA algorithm in detail elsewhere (Nunez-Iglesias et al., 2013), in this paper we focus on the design aspects of our implementation. We briefly present the Python application programming interface (API) and the command-line interface (CLI), before delving more deeply into particularly useful design choices, and finally discussing the current limitations of the library and future directions.

## 2. API

### 2.1. Python API

GALA belongs a class of segmentation algorithms called agglomerative algorithms, in which segments are formed by merging smaller segments. Other examples include mean agglomeration (Arbeláez et al., 2010), the graphical models of Andres et al. (2012a,b), and Learning to Agglomerate Superpixel Hierarchies (LASH) (Jain et al., 2011), which is most similar to GALA. Agglomerative methods begin with an initial fine-grained segmentation known as an *oversegmentation* or *superpixel map*. The superpixel approach allows a massive reduction in computational cost, enabling the use of more sophisticated algorithms in the agglomerative step. Additionally, it allows the use of different strategies to group pixels and regions, which may have very different properties (Ren and Malik, 2003).

GALA uses machine learning to obtain a merge priority function or policy, which dictates which pair of segments to merge next. It has three main prerequisites to learn this function:

- a *superpixel map* (or supervoxel), an initial fine-grained segmentation; and
- a *gold standard segmentation*, that represents the true segmentation of a training volume; and
- a pixel-level intensity map, which is optional but required for most features. This is usually the *probability of boundary* between segments, but can be other things, such as the probability of the pixel belonging to a glial cell, or to an image of a cat (Le et al., 2012). The map can even be the input image itself. Indeed, gala allows multi-channel pixel-level maps that are the concatenation of some or all of the above maps.

For the first requirement, we have used the watershed algorithm (Vincent and Soille, 1991), but other methods, such as SLIC (Achanta et al., 2012) would work. Gala itself contains an implementation of watershed, although for some parameter sets we just wrap the implementation in scikit-image, which is more efficient and works both in 2D and 3D. Again, the only requirement here is that the input volume is partitioned into some integer-labeled regions, and that these regions do not cross true segment boundaries, at least approximately. The algorithm that generates this initial oversegmentation is not important.

The second requirement is a completely segmented image to be used as ground truth. For neuronal EM images, we used ground truth segmentation generated with the open-source Raveler software (Olbris et al., in preparation), while a large ground truth body exists for natural images in the Berkeley Segmentation Data Set (BSDS) (Martin et al., 2001). For other images, such as 3D fluorescence microscopy images, generation of ground truth can be a laborious process. This has indeed become the rate-limiting step in a gala segmentation, so we are moving to eliminate this requirement so that only a subset of ground truth is needed (see discussion).

For the final requirement, we have used Ilastik (Sommer et al., 2011) in our own work on EM images, gPb (Maire et al., 2008) for natural images, or the probability maps provided by Ciresan et al. (2012) for the SNEMI3D challenge. Gala is agnostic about the origin of the input probability maps, both theoretically and in this implementation.

Given the above, we create a region adjacency graph, or RAG (implemented in gala.agglo.Rag) corresponding to the training superpixel and probability maps, and perform repeated training agglomerations of the superpixels while comparing against the ground truth (Rag.learn_agglomerate). This produces a training set, to which we can fit a classifier, which will then prioritize merges in a test volume to segment. These operations are illustrated in the following code snippets, which can be run from gala's tests/example-data directory.

First, we import the relevant gala modules and read in the data using the imio (image IO) module:

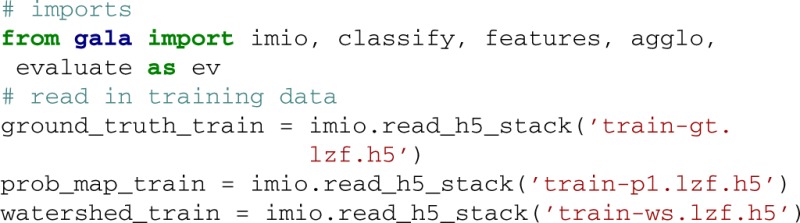



Next, we create a feature manager. These can be concatenated using the Composite manager. Managers are covered in more detail in section 3.2.

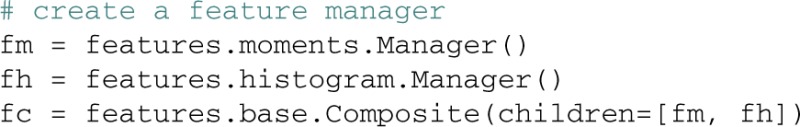



Using the feature manager, watershed oversegmentation, ground truth segmentation, and probability map, we can create a region adjacency graph g_train and obtain a (features, labels) training dataset.

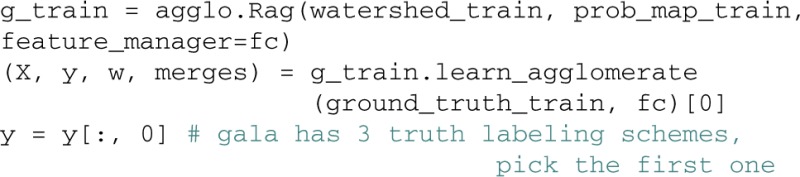



With the training dataset, we can train a classifier, using scikit-learn syntax. Indeed, any scikit-learn classifier can be used here.


rf = classify.DefaultRandomForest().fit(X, y)


By composing the feature map and the classifier, we obtain a policy: a function whose input is a graph and two nodes (representing segments) and whose output is a number in [0, 1].


learned_policy = agglo.classifier_probability(fc, rf)


This policy is then used to segment a test (previously unseen) volume. We agglomerate the superpixels until the classifier returns a merge probability of 0.5, which corresponds to even odds that the merge is a true or false merge. (This assumes a well-calibrated classifier, meaning that the output corresponds to the probability of a sample feature vector belonging to the “+1” class. Bostrom (2008) showed random forests to be reasonably well-calibrated.)

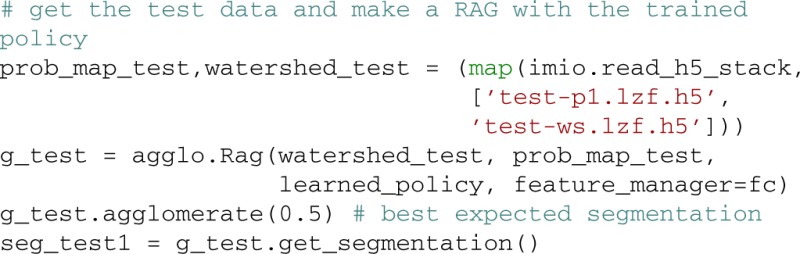



Because gala was created as research software, it implements a number of additional agglomerative segmentation algorithms, including mean boundary, oriented mean boundary (Arbeláez et al., 2010), median boundary, superpixel affinity learning (Ren and Malik, 2003) (which we also call “flat” learning), and LASH (Jain et al., 2011). These are invoked simply by using a different merge_priority_function keyword argument, or calling agglo.Rag.learn_agglomerate with different parameters.

The gala API presents a simple tool to obtain state of the art segmentations, and also allows the exploration of a complete set of hierarchical agglomerative segmentation strategies. Further, because the segmentation strategy is learned, it can be applied with very little modification to many different domains, as demonstrated by its success in natural image segmentation as well as two different kinds of EM data.

### 2.2. Segmentation evaluation module

One of the most generally reusable parts of the gala library is the evaluation module in gala/evaluate.py. It offers efficient implementations of edit distance, Rand Index (Rand, 1971), Adjusted Rand Index (Hubert and Arabie, 1985), Fowlkes-Mallows index (Fowlkes and Mallows, 1983), and Variation of Information (VI) (Meila, 2005).

Among these, we focused the most effort on the VI metric for its numerous advantages (Meila, 2005; Nunez-Iglesias et al., 2013). VI is an information theoretic measure to compare clusterings or segmentations. It precisely answers the following question: given the identity of a point in segmentation B, how much information, on average, will I need to know its identity in segmentation A, and vice-versa? If the two segmentations match perfectly, the answer is 0 bits: no additional information is required if the identity in A is the same as the identity in B in every case. Formally, it is the sum of the conditional entropy of A given B and the conditional entropy of B given A:

(1) VI(A,B)=H(A|B)+H(B|A)

When A is our automatic segmentation and B is the gold standard, *H*(*A*|*B*) measures the oversegmentation or false splits, and *H*(*B*|*A*) measures the undersegmentation or false merges.

The two components of VI are computed efficiently (*O*(*n*_pixels_) time complexity) with the evaluate.split_vi function.

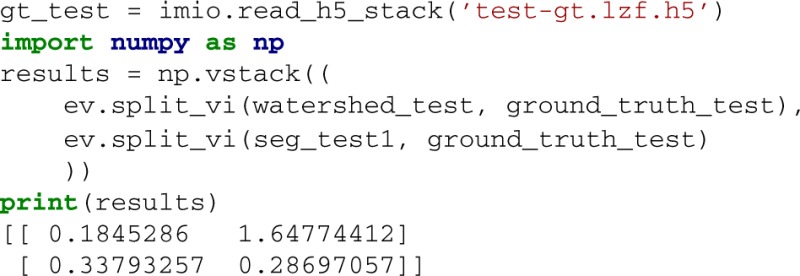



This is interpreted as follows: the undersegmentation VI of the watershed superpixels compared to the ground truth segmentation is 0.18 bits. That is, the average watershed basin will have slightly over 97% overlap with one ground truth body and 3% with another (since −0.97 log_2_(0.97) − 0.03log_2_(0.03) = 0.19 ≈ 0.185). In contrast, its oversegmentation VI is 1.64 bits, which means that most ground truth segments are split into more than 3 watershed pixels. (The conditional entropy of a perfect (1/3, 1/3, 1/3) split is 1.58 bits. A perfect 50-50 split has an entropy of 1 bit.)

**Figure 2:**
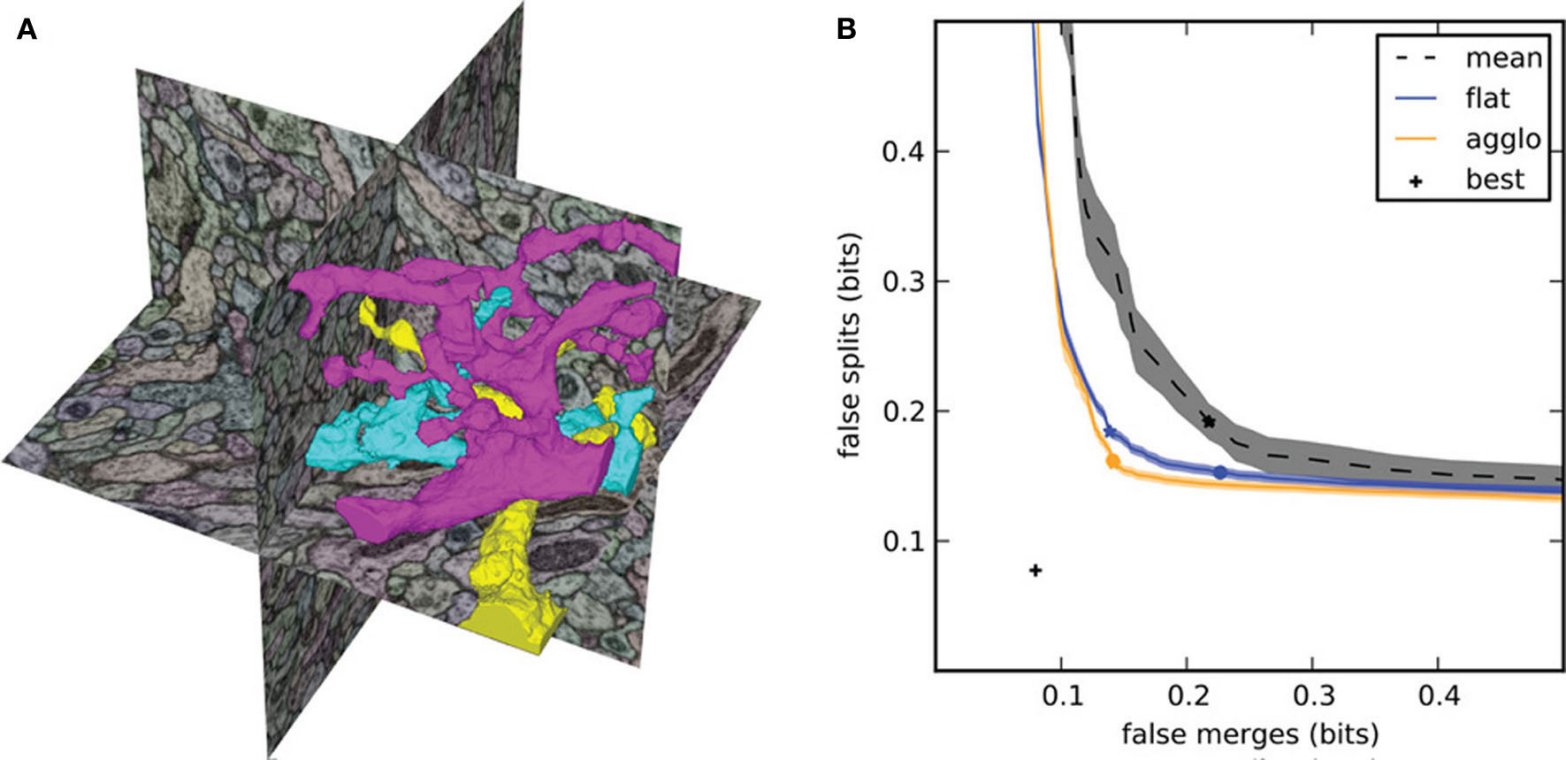
**Segmentation results for the focused ion beam scanning electron microscopy (FIBSEM) dataset of *Drosophila melanogaster* larva brain in Nunez-Iglesias et al. (2013). (A)** Example automatic segmentation of an octant of the dataset. The complex shapes of the three highlighted segments illustrate the difficulty of segmenting neuronal data. **(B)** Split VI plot from Nunez-Iglesias et al. (2013), showing superior segmentation accuracy by gala over competing agglomerative algorithms. Lower and to the left is better. The stars indicate the point of lowest VI, and the circles indicate the point at threshold 0.5. Shaded areas show standard error of the mean for *n* = 56 observations (“flat,” “agglo”) or *n* = 8 observations (“mean”). The point labeled “best” represents the VI of a perfect merging of the initial (imperfect) superpixels.

By computing the two conditional entropies at each segmentation threshold, we generate the split VI plot (Figure 2) that we introduced in Nunez-Iglesias et al. (2013), showing the tradeoff between oversegmentation and undersegmentation. In this plot, the x-axis is the undersegmentation conditional entropy, measuring false merges, and the y-axis is the corresponding oversegmentation measurement. Agglomerative segmentations begin somewhere close to the y-axis (lots of oversegmentation but very little undersegmentation). Then, a correct merge results in a downward move along the plot, while an incorrect merge causes a rightward move. The goal of a good segmentation algorithm, then, is to get as close as possible to (0, 0), a perfect match between automatic segmentation and ground truth.

This approach is in contrast to the commonly used plot of VI against segmentation threshold (see e.g., Andres et al., 2012b), which obscures the tradeoff information.

### 2.3. Command-line interface

In addition to the flexible Python library interface, we developed a set of command-line scripts to perform common gala functions, such as training and segmenting. This is the primary way of interacting with gala in a production environment. The scripts make use of the excellent argparse module, so usage can be determined by running the scripts with the -h or –help flags. Each option can be provided on the command line or through a JSON configuration file format.

## 3. Design highlights

In this section, we focus on a few design elements that we consider essential to gala's success.

### 3.1. N-dimensional array support

Many segmentation libraries assume 2D or 3D data, or provide separate functions for each (see, for example, Achanta et al., 2010, or many OpenCV functions).

We instead abstracted away the notion of a neighboring pixel (or voxel) with a get_neighbor_idxs function that depends only on the pixel coordinates, the shape of the array, and a connectivity parameter. As all operations in our algorithm depend only on the definition of the local neighborhood, this abstraction made gala dimension-agnostic. This allowed us to produce segmentations of the Berkeley segmentation dataset (Martin et al., 2001) and a 3D EM dataset from the same code base.

A great many algorithms in computer vision can be parameterized by neighboring voxels. Thus, we encourage developers to write these using n-dimensional logic from the start to increase the range of applications of their software. The numpy library's excellent ndarray object was essential for our n-dimensional support, making gala a prime example of the success of the Python ecosystem for scientific computing.

### 3.2. Feature managers and feature caches

An example of a critical feature when determining the probability that two segments should be merged is the average pixel-level probability of boundary (Ren and Malik, 2003). As segments are merged, the shared surface between them and their common neighbors increases, thereby making the average a more reliable estimate of the true probability of a boundary. Recomputing the mean from scratch, however, results in quadratic time complexity, because we are repeatedly iterating over the same pixels after each merge. Therefore, a more efficient strategy is to cache a sum of the pixel probabilities and the count of pixels examined. Then, when two boundaries are combined, their probability sums are added together, as are their counts, and the new average probability can be computed by dividing the new sum by the new count, in constant time. This caching turns a quadratic operation into a linear one.

The above concept can be generalized to a large class of features. We found that, in many cases, caching intermediate computations dramatically improved the time complexity of feature computation. For example, to compute the standard deviation of the pixel probabilities, we must cache the sum of the squared pixel probabilities, along with simple sum and counts. To compute a histogram, we cache the unnormalized histogram, and each bin is summed when two boundaries are combined.

We therefore devised a single class, which we call a feature manager, that is responsible for defining the cached values, and for computing the feature vector from the cached values. This has enabled less obvious features, including, for example, some based on the convex hull of the segment. The convex hull feature manager stores as a cache the convex hull of each node. When two nodes are merged, the resulting convex hull can be computed faster by starting from the two initial hulls, rather than from the newly formed segment, since these have fewer vertices than the segments themselves. The manager then uses the hull to compute features such as segment convexity, by comparing the volume of the convex hull to the volume of the segment.

We have strived to make it easy to develop new feature managers, which will be useful as new, more sophisticated 3D segmentation features are developed (Bogovic et al., 2013). A GitHub pull request creating a new manager can be found at: https://github.com/jni/ray/pull/51

### 3.3. Classifier abstraction

Given the vast heterogeneity of our initial feature space, we wished to use a random forest (RF) as our classifier of choice. scikit-learn, the present gold-standard in machine learning Python libraries, did not contain a RF implementation when we started building gala. We therefore decided to use Vigra, a C++ image analysis and machine learning library with Python bindings. However, we recognized that a cross-compatible interface across libraries would allow rapid testing of various machine learning techniques. We therefore built a wrapper around Vigra to match scikit-learn's estimator interface, particularly the fit(), predict(), and predict_proba() methods.

Because of this, it is trivial to try different classifiers for the learning and agglomeration steps of gala. In particular, we have been able to use the recently vastly improved RandomForestClassifier from scikit-learn version 0.14 with no code changes.

In short, by using established interfaces, we were able to future-proof our software. We recommend that anyone looking to build software in the Python ecosystem take a long look at related libraries to match interfaces as closely as possible.

## 4. Discussion

Although we have discussed many of gala's strengths and its positive design aspects, it does currently have limitations, which we describe here, along with potential fixes.

### 4.1. Complete gold standard requirement

As currently implemented, gala requires a fully segmented volume from which to learn. In our experiments, this has become the major bottleneck when starting segmentations on new data. Therefore, a priority in gala development going forward is the ability to mask volumes so that partial ground truth can be used.

### 4.2. Memory and time inefficiency

Gala's implementation, based on NetworkX, is slow and has a high memory footprint. However, many improvements are within easy reach.

Firstly, we currently store feature caches and compute feature vectors as separate arrays. This results in a huge time overhead for large graphs due to memory allocation, and also in memory usage because of the dictionaries required to store all the separate arrays. However, because we are performing a hierarchical agglomeration, we know that the number of nodes and edges is bounded by twice the initial number. Therefore, we can pre-allocate an initial array of shape (2 * n_nodes, cache_size) for the node feature caches, and similarly for the edges, and use an incremental indexing scheme to keep track of which node or edge in the hierarchy uses which row of the array.

Additionally, the graph currently stores indices to the voxels comprising each node and boundary, which is unnecessary. Space and time can be saved by keeping only a single voxel and rebuilding nodes using a flood fill.

Finally, we chose the heavy Graph class of the NetworkX library for its flexibility and fast node addition and removal. However, this is ultimately unnecessary: we can store the original supervoxel graph using a much more efficient structure, such as scipy.sparse.csc_graph, and maintain a merge tree. The graph at any level of the hierarchy can be rapidly constructed from this.

## 5. Conclusions

Like most academic software, gala is a mixture of new algorithms, some good design, and a variety of questionable decisions left over from a time of different priorities. We wrote this description in the hope that the existing and future functionality, the better parts of the software, and the lessons learned will be of value to the wider research community. We particularly emphasize that Don Knuth's famous maxim that “premature optimization is the root of all evil” (Knuth, 1974) should not be taken to extremes: in our case, this has led to time and memory performance issues that have been difficult to resolve. Still, gala's success in segmenting not only the isotropic EM volume for which it was designed (Glasner et al., 2011; Nunez-Iglesias et al., 2013), but also the BSDS natural image dataset and the SNEMI3D anisotropic EM dataset, suggests that it will be useful for some time to come. In future work, we will explore the potential of gala to segment other kinds of neuronal data, including 3D light microscopy data and 3D+t neuronal activity data.

### Conflict of interest statement

The authors declare that the research was conducted in the absence of any commercial or financial relationships that could be construed as a potential conflict of interest.

## References

[B1] Achanta R. Shaji A. Smith K. Lucchi A. Fua P. (2012). SLIC superpixels compared to state-of-the-art superpixel methods. IEEE Trans. Pattern Anal. Mach. Intell. 34, 2274–2282. 10.1109/TPAMI.2012.120 22641706

[B2] Achanta R. Shaji A. Smith K. Lucchi A. Fua P. Süsstrunk S. (2010). SLIC Superpixels. Available online at: http://ivrg.epfl.ch/research/superpixels 10.1109/TPAMI.2012.120 22641706

[B3] Andres B. Koethe U. Kroeger T. Helmstaedter M. Briggman K. L. Denk W.. (2012a). 3D segmentation of SBFSEM images of neuropil by a graphical model over supervoxel boundaries. Med. Image Anal. 16, 796–805. 10.1016/j.media.2011.11.004 22374536

[B4] Andres B. Kroeger T. Briggman K. L. Denk W. Korogod N. Knott G. (2012b). Globally optimal closed-surface segmentation for connectomics, in ECCV (Florence), 778–791.

[B5] Arbeláez P. Maire M. Fowlkes C. Malik J. (2010). Contour detection and hierarchical image segmentation. IEEE Trans. Patt. Anal. Mach. Intell. 33, 898–916. 10.1109/TPAMI.2010.161 20733228

[B6] Arganda-Carreras I. Seung S. H. Vishwanathan A. Berger D. (2013). SNEMI 3D: 3D Segmentation of Neurites in EM Images. Available online at: http://brainiac2.mit.edu/SNEMI3D/.

[B7] Bogovic J. A. Huang G. B. Jain V. (2013). Learned versus hand-designed feature representations for 3d agglomeration. E-print: arXiv:1312.6159.

[B8] Bostrom H. (2008). Calibrating random forests, in Machine Learning and Applications, 2008. ICMLA'08. Seventh International Conference on (San Diego, CA), 121–126.

[B9] Cardona A. Saalfeld S. Preibisch S. Schmid B. Cheng A. Pulokas J.. (2010). An integrated micro- and macroarchitectural analysis of the Drosophila brain by computer-assisted serial section electron microscopy. PLoS Biol. 8:e1000502. 10.1371/journal.pbio.1000502 20957184 PMC2950124

[B10] Chklovskii D. B. Vitaladevuni S. Scheffer L. K. (2010). Semi-automated reconstruction of neural circuits using electron microscopy. Curr. Opin. Neurobiol. 20, 667–675. 10.1016/j.conb.2010.08.002 20833533

[B11] Ciresan D. Giusti A. Gambardella L. M. Schmidhuber J. (2012). Deep neural networks segment neuronal membranes in electron microscopy images, in Proceedings of Neural Information Processing Systems (Lake Tahoe, NV), 2852–2860.

[B12] Fowlkes E. B. Mallows C. L. (1983). A method for comparing two hierarchical clusterings. J. Am. Stat. Assoc. 78, 553–569. 10.1080/01621459.1983.10478008

[B13] Glasner D. Hu T. Nunez-Iglesias J. Scheffer L. Xu S. Hess H. (2011). High resolution segmentation of neuronal tissues from low depth-resolution EM imagery, in EMMCVPR '11 (St. Petersburg), 1–12.

[B14] Helmstaedter M. (2013). Cellular-resolution connectomics: challenges of dense neural circuit reconstruction. Nat. Methods 10, 501–507. 10.1038/nmeth.2476 23722209

[B15] Helmstaedter M. Briggman K. L. Denk W. (2011). High-accuracy neurite reconstruction for high-throughput neuroanatomy. Nat. Neurosci. 14, 1081–1088. 10.1038/nn.2868 21743472

[B16] Helmstaedter M. Briggman K. L. Turaga S. C. Jain V. Seung H. S. Denk W. (2013). Connectomic reconstruction of the inner plexiform layer in the mouse retina. Nature 500, 168–174. 10.1038/nature12346 23925239

[B17] Hubert L. Arabie P. (1985). Comparing partitions. J. Classif. 2, 193–218. 10.1007/BF01908075

[B18] Jagadeesh V. Anderson J. Jones B. Marc R. Fisher S. Manjunath B. S. (in press). Synapse classification and localization in electron micrographs. Patt. Recogn. Lett. 10.1016/j.patrec.2013.06.001

[B19] Jain V. Turaga S. C. Briggmann K. L. Helmstaedter M. N. Denk W. Seung H. S. (2011). Learning to agglomerate superpixel Hierarchies, in Advances in Neural Information Processing Systems (Granada), 24.

[B20] Kaynig V. Vazquez-Reina A. Knowles-Barley S. Roberts M. Jones T. R. Kasthuri N. (2013). Large-Scale automatic reconstruction of neuronal processes from electron microscopy images. arXiv:1303.7186. 10.1016/j.media.2015.02.001 PMC4406409 25791436

[B21] Knuth D. (1974). Structured programming with go to statements. ACM J. Comput. Surv. 6, 268.

[B22] Kreshuk A. Straehle C. N. Sommer C. Koethe U. Cantoni M. Knott G.. (2011). Automated detection and segmentation of synaptic contacts in nearly isotropic serial electron microscopy images. PLoS ONE 6:e24899. 10.1371/journal.pone.0024899 22031814 PMC3198725

[B23] Le Q. Ranzato M. Monga R. Devin M. Chen K. Corrado G. (2012). Building high-level features using large scale unsupervised learning, in International Conference in Machine Learning (Edinburgh).

[B24] Liu T. Jones C. Seyedhosseini M. Tasdizen T. (2014). A modular hierarchical approach to 3D electron microscopy image segmentation. J. Neurosci. Methods 226, 88–102. 10.1016/j.jneumeth.2014.01.022 24491638 PMC3970427

[B25] Liu T. Jurrus E. Seyedhosseini M. Ellisman M. Tasdizen T. (2012). Watershed merge tree classification for electron microscopy image segmentation, in Pattern Recognition, ICPR 2012 (Tsukuba), 133–137. PMC4256108 25485310

[B26] Maire M. Arbelaez P. Fowlkes C. Malik J. (2008). Using contours to detect and localize junctions in natural images, in Computer Vision and Pattern Recognition, 2008. CVPR 2008. IEEE Conference on (Anchorage, AK), 1–8.

[B27] Martin D. Fowlkes C. Tal D. Malik J. (2001). A database of human segmented natural images and its application to evaluating segmentation algorithms and measuring ecological statistics, in Proceedings of the 8th International Conference Computer Vision, Vol. 2 (Vancouver, BC), 416–423.

[B28] Meila M. (2005). Comparing clusterings: an axiomatic view, in Proceedings of the 22nd International Conference on Machine learning, ICML '05 (New York, NY: ACM), 577584.

[B29] Nunez-Iglesias J. Kennedy R. Parag T. Shi J. Chklovskii D. B. (2013). Machine learning of hierarchical clustering to segment 2D and 3D images. PLoS ONE 8:e71715. 10.1371/journal.pone.0071715 23977123 PMC3748125

[B31] Rand W. M. (1971). Objective criteria for the evaluation of clustering methods. J. Am. Stat. Assoc. 66, 846–850. 10.1080/01621459.1971.10482356

[B32] Ren X. Malik J. (2003). Learning a classification model for segmentation, in ICCV 2003: 9th International Conference on Computer Vision, Vol. 1 (Beijing), 10–17.

[B33] Sommer C. Straehle C. Koethe U. Hamprecht F. A. (2011). ilastik: interactive learning and segmentation toolkit, in 8th IEEE International Symposium on Biomedical Imaging (ISBI 2011) (Chicago, IL), 230–233. 10.1109/ISBI.2011.5872394

[B34] Takemura S.-Y. Bharioke A. Lu Z. Nern A. Vitaladevuni S. Rivlin P. K.. (2013). A visual motion detection circuit suggested by Drosophila connectomics. Nature 500, 175–181. 10.1038/nature12450 23925240 PMC3799980

[B35] Vincent L. Soille P. (1991). Watersheds in digital spaces: an efficient algorithm based on immersion simulations. PAMI 13, 583–598. 10.1109/34.87344

